# Sex Differences of Vitamin D Status across BMI Classes: An Observational Prospective Cohort Study

**DOI:** 10.3390/nu11123034

**Published:** 2019-12-12

**Authors:** Giovanna Muscogiuri, Luigi Barrea, Carolina Di Somma, Daniela Laudisio, Ciro Salzano, Gabriella Pugliese, Giulia de Alteriis, Annamaria Colao, Silvia Savastano

**Affiliations:** Dipartimento di Medicina Clinica e Chirurgia, Unit of Endocrinology, Federico II University Medical School of Naples, Via Sergio Pansini 5, 80131 Naples, Italy; giovanna.muscogiuri@gmail.com (G.M.); cdisomma@unina.it (C.D.S.); daniela.laudisio@libero.it (D.L.); cirosalzano89@gmail.com (C.S.); robiniapugliese@gmail.com (G.P.); dealteriisgiulia@gmail.com (G.d.A.); colao@unina.it (A.C.); sisavast@unina.it (S.S.)

**Keywords:** vitamin D, obesity, bioelectrical impedance analysis, gender differences, vitamin D supplementation, nutritionist

## Abstract

Growing evidence reported that vitamin D deficiency is a common finding in obesity. Vitamin D status also seems to be sex-related, although little is known regarding this association. Therefore, the aim of this study was to investigate the sex-related differences of serum 25OH vitamin D (25OHD) concentrations across body mass index (BMI) classes and, if there were any differences, whether they could be explained by sex-related differences in body composition. We enrolled 500 subjects (250 males, age 37.4 ± 11.8 years; 250 females, age 36.6 ± 11.8 years). Body composition was assessed by bioelectrical impedance analysis (BIA) phase-sensitive system. Serum 25OHD concentration was quantified by a direct, competitive chemiluminescence immunoassay. Vitamin D deficiency was defined as a serum 25OHD concentrations < 20 ng/mL (50 nmol/L). Stratifying the sample population according to sex and BMI categories, 25OHD concentrations were significantly higher in males compared to females in all BMI classes and decreased along with the increase of BMI values. Females with vitamin D deficiency had higher fat mass (FM) % compared to males with vitamin D deficiency. The 25OHD concentrations inversely correlated with FM % in both sexes. In a multiple regression analysis model, sex, FM %, and BMI were predictive factors of 25OHD concentration. In conclusion, our study suggests that 25OHD concentrations were lower in females than males across all BMI categories. Given the tight correlation between 25OHD concentrations and FM %, it can be hypothesized that the lower 25OHD concentrations in females than males can be explained by the fact that females have a higher amount of fat than males.

## 1. Introduction

The main physiologic role of vitamin D is to regulate calcium and phosphorus homeostasis and to promote bone homeostasis [1]. However, accumulating evidence from animal and human studies suggest that vitamin D may also be important for a variety of non-skeletal actions and, thus, vitamin D deficiency has been associated with a host of chronic diseases such as cardiovascular diseases, cancer, type 1 diabetes (T1DM), and type 2 diabetes (T2DM) [2]. The increased interest in the pleiotropic effects of vitamin D and the high prevalence of vitamin D deficiency in the general healthy population has generated very high interest in the vitamin D research field among researchers, clinicians, and the lay public. In particular, vitamin D deficiency is a common finding in obesity [3,4,5]. Indeed, data coming from The National Health and Nutrition Examination Survey (NHANES) 2005 to 2006 provide information regarding the prevalence of vitamin D deficiency in people with obesity and at normal weight. The 2005 to 2006 NHANES included certain subgroups of the US population, including low-income persons, older adults aged 60 years or older, African Americans, and Mexican Americans, to provide a more in-depth snapshot of these population groups. A total of 12,862 individuals were sampled in the 2005 to 2006 NHANES. Among the sampled individuals, 10,348 (80.5%) participated in the interview and 8306 (65%) provided valid data on vitamin D measurement. The findings of this study reported that the prevalence of vitamin D deficiency (vitamin D concentration < 20 ng/mL) has been reported to be higher (53.8%) in subjects with obesity compared to normal weight (33%) [6]. Body fat may represent a reservoir for vitamin D storage, reducing its bioavailability [7]. An inverse correlation between serum 25OHD concentration and magnitude of weight loss in people with obesity after bariatric surgery confirms this theory [8]. Indeed, large accrual in adipose tissue depot implies that vitamin D3 could not be appropriately released into the general circulation to support serum 25OHD concentrations. However, the sequestration and the inappropriate storage of vitamin D in adipose tissue seems to not fully explain the low vitamin D status of obesity, as once the values in obesity are adjusted for body size, there is no longer a difference in 25OHD concentrations between normal and obese individuals. Convincing data suggest that the low vitamin D status of obesity could be simply the consequence of the volumetric dilution of ingested or cutaneously synthesized vitamin D3 in the large FM of obese patients [9]. A predisposing factor to vitamin D deficiency in people with obesity might also be found in low dietary vitamin D intake and insufficient physical activity (resulting in limited sun exposure). As a result of vitamin D deficiency, secondary hyperparathyroidism is a common finding in people with obesity, and it is often secondary to vitamin D deficiency [10,11]. In turn, both vitamin D deficiency and increased parathyroid hormone (PTH) concentration have been reported to worsen glucose metabolism thus contributing to *obesity-related* glucose derangements [3]. Further, the increase in PTH concentration may increase 1α,25(OH)2D3 concentration that, in turn, could exert a negative feed-back on hepatic synthesis of 25OHD thus contributing to worsen vitamin D deficiency [12]. Johnson et al. [13] reported that morbidly obese Norwegian men seeking weight loss treatment have been found to have significantly higher odds of vitamin D deficiency than women with comparable BMI values. Conversely, in a study performed in Southern Italy, the prevalence of hypovitaminosis D, defined by a concentration of 25OHD lower than 30 nmol/L, was 27.8% in winter and 3.4% in summer in young healthy females while young healthy males subjects did not display hypovitaminosis D throughout the year [14].

Thus, the objectives of our study were (1) to investigate the sex differences in vitamin D status across BMI classes and, (2) if any differences exist, whether it could be explained by sex-related differences in body composition. 

## 2. Material and Methods

### 2.1. Study Design 

The experimental design of the study was an observational prospective cross-sectional study.

### 2.2. Setting

The study was performed at the Department of Clinical Medicine and Surgery, Endocrinology Unit, University Federico II, Naples (Italy), from 13 October 2016 to 25 March 2019. The study was carried out taking into account the Code of Ethics of the World Medical Association (Declaration of Helsinki) for experiments involving humans. The protocol was formally approved by the Ethical Committee of the University of Naples “Federico II” Medical School (*n*. 173/16). Every enrolled subject provided informed consent after a thorough explanation of the protocol.

### 2.3. Participants

A sample of 571 adult Caucasians subjects was consecutively recruited among patients of our outpatient clinic, hospital volunteers, and employees living in the Naples metropolitan area (latitude 40°49’N; elevation, 17 m). We enrolled female subjects that were non-pregnant and non-lactating. A full medical history, including drug use, was collected. 

**Figure 1:**
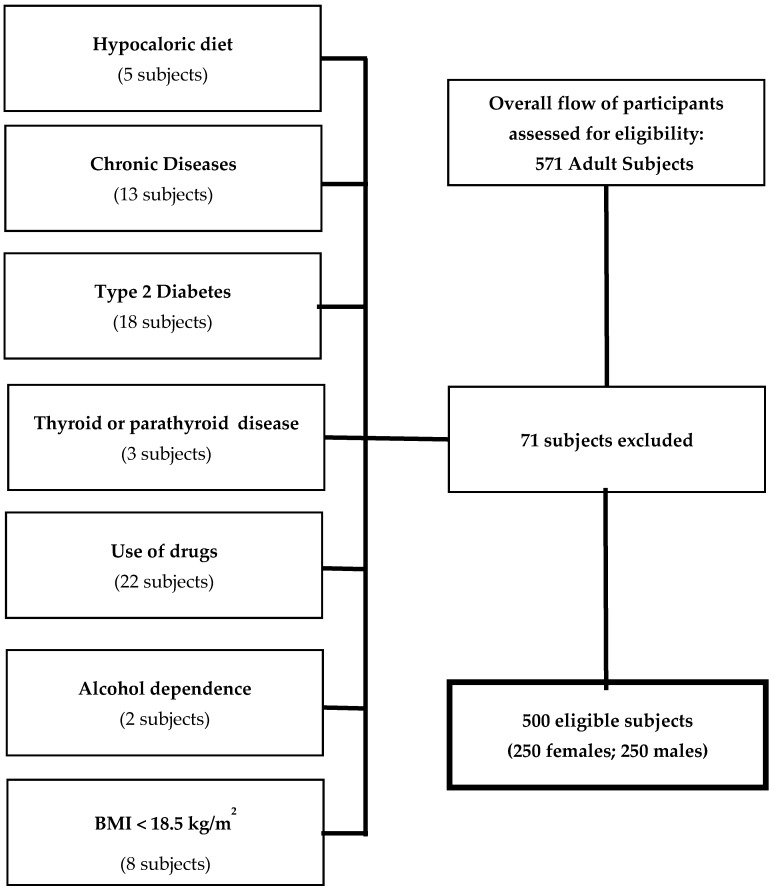
The flowchart of study subjects.

In order to increase the homogeneity of the subject samples, we included only adults of both gender with the following criteria of exclusion (Figure 1):

- Hypocaloric diet in the last three months (5 subjects);
- Chronic diseases that could interfere fluid homeostasis, such as liver or renal chronic diseases, cancer, acute or chronic inflammatory diseases (13 subjects);
- Altered concentration of serum creatinine, serum calcium, or albumin;
- Presence of T2DM (defined by criteria of the American Diabetes Association as follows: basal plasma glucose level ≥ 126 mg/dL on two occasions, or glycated hemoglobin (HbA1c) ≥ 6.5% (≥48 mmol/mol) on two occasions, or both at the same time. Participants on antidiabetic medication were considered to have T2DM (18 subjects);
- Uncontrolled thyroid or parathyroid disease (3 subjects);
- Current therapy with calcium, vitamin D supplementation or osteoporosis therapies, anti-inflammatory drugs, statin, and other hypolipidemic agents (22 subjects);
- Alcohol dependence diagnosed based on the Diagnostic and Statistical Manual of Mental Disorders (DSM)-V diagnostic criteria (2 subjects);
- Pacemakers or defibrillators which could potentially interfere with BIA assessment;
- Patients having a BMI lower than 18.5 kg/m^2^ (8 subjects).

### 2.4. Measurements

#### 2.4.1. Anthropometric Measurements 

Measurements were performed in the morning, after an overnight fast. The anthropometric assessment was performed following standard criteria by the same nutritionist. The subjects were recommended to dress in light clothes and to remove shoes during the assessment [15,16,17]. The BMI (weight (kg) divided by height squared (m^2^), kg/m^2^) was calculated after measuring weight and height. A wall-mounted stadiometer (Seca 711; Seca, Hamburg, Germany) was used to measure height while a calibrated balance beam scale (Seca 711; Seca, Hamburg, Germany) was used to assess weight. The degrees of obesity were established according to the World Health Organization’s (WHO) criteria: BMI: 18.5–24.9 kg/m^2^, normal weight; BMI: 25.0–29.9 kg/m^2^, overweight; BMI: 30.0–34.9 kg/m^2^, grade I obesity; BMI: 35.0–39.9 kg/m^2^, grade II obesity; BMI ≥ 40.0 kg/m^2^, grade III obesity [18]. 

#### 2.4.2. Bioelectrical Impedance Analysis

The BIA phase-sensitive system (800 µA, 50 kHz; BIA 101 RJL, Akern Bioresearch, Florence, Italy) was used by experienced observers to assess body composition [19] as previously reported [17,20,21,22]. We performed the exam as suggested by the European Society of Parental and Enteral Nutrition (ESPEN) [23]. Electrodes were placed on the hand and the ipsilateral foot according to Kushner (1992) [19]. We used the following formula: phase angle, PhA (°, degrees) = arctangent reactance (Xc)/resistance (R) × (180/π)) to calculate PhA obtained from conditions under 50 kHz.

#### 2.4.3. Assay Methods

We stored the blood samples, collected in the morning after an overnight 8 h fast, at −80 °C until they were processed. Serum 25OHD concentrations were measured with chemiluminescence (Liaison, DiaSorin, Saluggia, Italy). The analytical measurement range of detection was 4–150 ng/mL, whereas the intra-assay coefficients of variation (CVs) were 5.4%, 2.8%, and 4.7%, and the inter-assay CVs were 10.1%, 4.8%, and 7.9% for low, medium, and high points of the standard curve, respectively, as previously reported [16,24,25].

Vitamin D deficiency was defined as a serum 25OHD concentrations < 20 ng/mL (50 nmol/L), insufficiency between 21 and 29 ng/mL (from 52.5 to 72.5 nmol/L), and normal concentrations ≥ 30 ng/mL (75 nmol/L) [26] as previously reported [16,25].

#### 2.4.4. Bias

The biases of our study were the following: (1) the data were not adjusted for seasonal variation and this was due to the fact that the subjects were enrolled in the very same season; (2) we did not adjust the data for sun exposure; however, we enrolled people living in the same Metropolitan area and with the same similar lifestyle habits regarding sun exposure; (3) we did not correct the data for vitamin D dietary intake; however, we excluded people taking vitamin D supplements.

#### 2.4.5. Study Size

The sample size was determined using the software ClinCalc tool (www.clincalc.com) based on the results from Carnevale et al. [14], considering 25OHD concentration as the main variable. A statistical power (1-β) of 95% and a level of significance (α) of 5% were considered which resulted in a sample of 52 subjects as the necessary number for this study.

#### 2.4.6. Statistical Analysis

The data distribution was evaluated by the Kolmogorov–Smirnov test and the abnormal data were normalized by logarithm. Skewed variables were back-transformed for presentation in tables and figures. Baseline descriptive statistics, including means and standard deviations for continuous characteristics and frequencies and percentages for categorical characteristics, were calculated. Similarly, descriptive statistics for all primary and secondary outcome measures at all time points in the study were also calculated. The differences between males and females in terms of age, anthropometric characteristics, 25OHD concentration, and body composition parameters were analyzed by Student’s paired *t*-test or ANOVA followed by the Bonferroni post-hoc test, while the chi-square (χ^2^) test was used to determine the significance of differences in frequency distribution among BMI classes (normal weight, overweight, grade I obesity, grade II obesity, and grade III obesity) and 25OHD categories (deficiency, insufficiency, and normal). Pearson *r* correlation coefficients were used in order to investigate correlations among 25OHD concentration with age, anthropometric measurements and body composition parameters after adjusting for BMI and FM. In addition, a multiple linear regression analysis model (stepwise method), expressed as *R*^2^, Beta (β) and *t*, with 25OHD concentration as dependent variables were used to estimate the predictive value of gender, BMI, and FM % on 25OHD concentration. Data were collected and analyzed using the MedCalc^®^ package (version 12.3.0 1993–2012, Mariakerke, Belgium). 

## 3. Results

### 3.1. Participants

The study population consisted of 500 participants: 250 males and 250 females. 

### 3.2. Descriptive Data

Age, anthropometric characteristics, and vitamin D of the study population are reported according to sex in Table 1. 

Although both males and females had comparable values of BMI, 25OHD concentrations were significantly higher in males than females, although both of them had vitamin D deficiency. The percentage of subjects with vitamin D insufficiency were significantly lower in males compared to females, while the percentage of subjects with vitamin D sufficiency was higher in males compared to females. Although there was no significant differences regarding the percentage of subjects with vitamin D deficiency between males and females, we found a trend toward a higher percentage of subjects with vitamin D deficiency in females.

**Figure 2:**
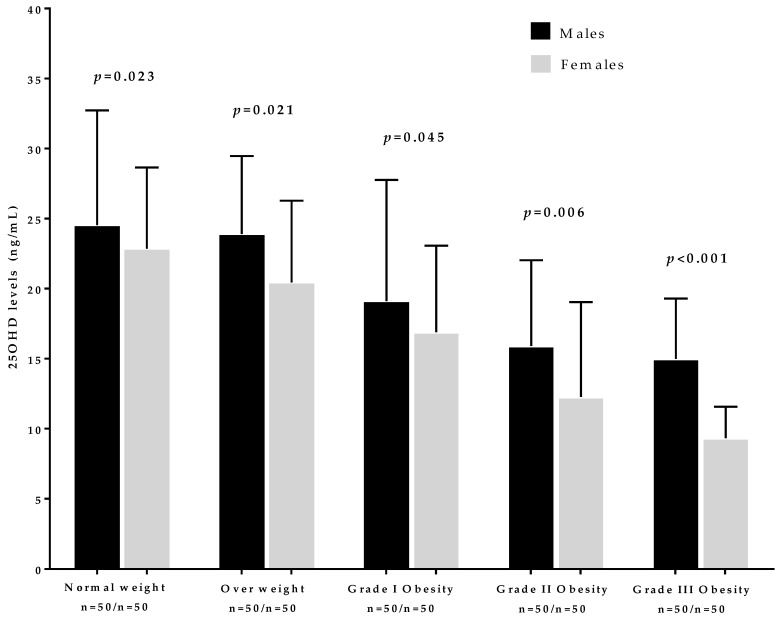
The 25OHD concentration in the population study across BMI categories according to sex. The 25OHD concentrations were found to be significantly higher in males than females in each BMI category. A *p*-value in bold type means a significant difference (*p* < 0.05).

Figure 2 reports 25OHD concentrations in the population study across BMI categories according to sex. The 25OHD concentrations were significantly higher in males compared to females across all BMI classes. Moreover, 25OHD concentrations had a trend towards lower 25OHD concentrations as BMI increased.

In Table 2, we report all body composition parameters (FM, free fat mass, FFM; total body water, TBW; extra-cellular water, ECW; and intra-cellular water, ICW) according to sex. Significant higher values of R (Ω), FM (%), and ECW (%) were found in females compared to males, while lower values of Xc (Ω), FFM (%), TBW (%), and ICW (%) were found in females compared to males.

**Figure 3:**
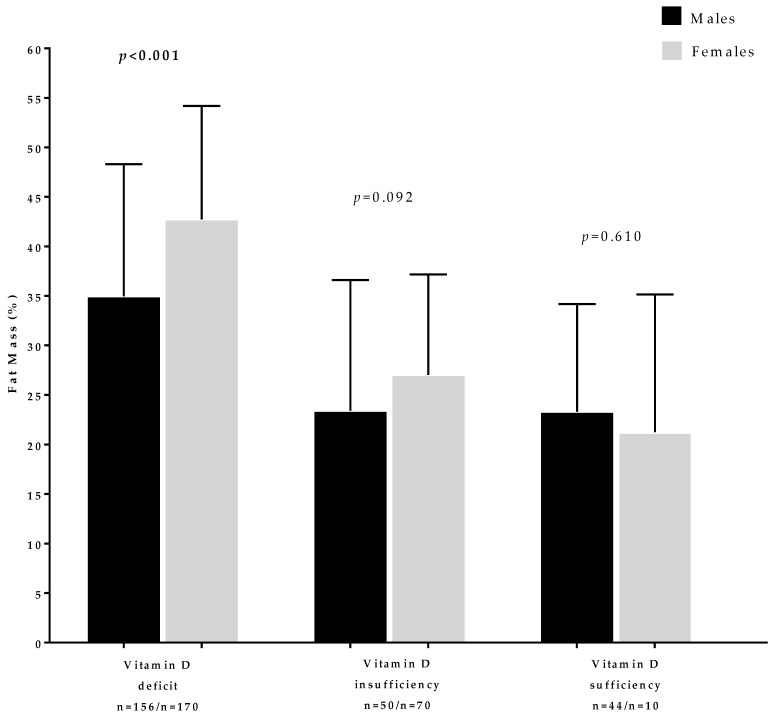
The fat mass % in the population study across 25OHD categories according to sex. Vitamin D deficiency was defined as a serum concentration of 25OH vitamin D < 20 ng/mL (50 nmol/L), insufficiency between 21 and 29 ng/mL (from 52.5 to 72.5 nmol/L), and normal concentration ≥ 30 ng/mL (75 nmol/L). A *p*-value in bold type means a significant difference (*p* < 0.05).

Figure 3 reports the FM % in the population study across vitamin D categories according to sex. Females with vitamin D deficiency have been found to have significantly higher FM % than males (*p* < 0.001). Moreover, we found a trend toward higher FM % in females compared to males in both vitamin D insufficiency and sufficiency categories.

### 3.3. Impact of Sex Difference on Vitamin D Relationship with Age and Body Composition Parameters

The correlations among 25OHD concentrations, age, BMI, and body composition parameters assessed by BIA are reported in Table 3. Apart from age, 25OHD concentrations showed significant and very strong negative correlations with BMI, FM%, and ECW (Lt), while it significantly and very strongly positively correlated to Xc (Ω), FFM%, TBW (Lt), and ICW (Lt) in females. We did not find a correlation between 25OHD concentration and R (Ω) in females. In males, we found that 25OHD concentration significantly and very strongly negatively correlated with BMI, R (Ω), FM %, and ECW (Lt), while it significantly and very strongly positively correlated to FFM %, TBW (Lt), ICW (Lt), and Xc (Ω).

To compare the relative predictive power of sex, FM %, and BMI on 25OHD concentration, we performed a multiple linear regression analysis using a model that included as independent variables sex, BMI, and FM %, and 25OHD as a dependent variable. Using this model, FM % was entered at the first step (*p* < 0.001), followed by BMI and sex. Results are reported in Table 4. 

## 4. Discussion

Study findings revealed a lower 25OHD concentrations in females compared to males. Moreover, female subjects with vitamin D deficiency had higher FM % compared to males with vitamin D deficiency. In both sexes, 25OHD concentrations were inversely related to BMI, FM %, and ECW, and they were directly related to Xc (Ω), FM %, TBW (Lt), and ICW. A positive correlation between R (Ω) and 25OHD concentrations were found only in males.

As shown by the results of the multivariate analysis, sex, BMI and FM were predictive factors of 25OHD concentrations. As already reported, FM represents a reservoir of 25OHD and its metabolites [7,27] thus decreasing circulating 25OHD concentration. In agreement with this finding, Blum et al. [27] carried out a cross-sectional study in subjects with severe obesity, reporting an inverse association between serum vitamin D3 concentrations and the mean vitamin D3 concentration in subcutaneous fat tissue samples thus supporting the long-standing concept that fat tissue traps serum vitamin D resulting in a decrease of circulating 25OHD concentration [27]. In our study, females were found to have lower 25OHD concentrations than males with similar BMI, and this was probably due to the more FM in females than males. On average, men have 10%–15% less fat content than women with the same BMI [28,29] thus having a smaller reservoir to sequestrate vitamin D [7]. Although we found a significant difference in terms of FM % between males and females with vitamin D deficiency, we only found a trend of difference between the genders in subjects with vitamin D insufficiency and normal vitamin D concentration without reaching statistical significance. This could be due to the fact that in vitamin D insufficiency and sufficiency groups, there were more lean subjects than the vitamin D deficiency group. It might be that the difference of FM % between the sexes in normal weight conditions is not enough to determine a different sex-related impact on vitamin D status. In addition to sex-differences in terms of vitamin D sequestrating property, another cause of sex-related difference of 25OHD concentration in females could be identified in sex different meal patterns. Indeed, it has been reported that females have a lower intake of fish that is currently considered the main dietary source of vitamin D [30]. Further, female sex was associated with more frequent extensive sunscreen use compared to males—a detriment to sun exposure [31,32]. A different action of sex hormones on vitamin D synthesis/breakdown could also be hypothesized. Total 25OHD concentrations have been reported to be higher in women who take oral contraceptives containing estrogen [33], and this could be explained by an estrogen-related increased hepatic hydroxylation of vitamin D [34] and vitamin D binding protein concentration in circulation [35]. However, this link was not confirmed in young women not using hormonal contraceptives, where a per increase of 10 nmol/L of 25OHD concentration led to a decrease in estradiol by a factor of 3% [36]. Also, total and free testosterone concentrations have been reported to positively correlate with 25OHD concentrations in healthy Korean men [37]. Daily supplementation with vitamin D in overweight healthy men for 12 months increased both serum 25OHD and testosterone concentration compared with administration of a placebo [38]. However, the bias of this intervention study was mainly due to the small sample size and to the participation of men to a diet program for weight reduction. The administration of 20,000 IU of vitamin D3/week for 12 weeks did not result in an increase of total testosterone in both healthy [39] and men with low testosterone [40].

The strengths of our study include the prospective collection of data in a large, homogenous population of individuals with different BMI categories and the assessment of body composition that allow us to partially explained the sex difference in 25OHD concentration. We did not adjust data for seasonal variations, but in order to minimize this bias, we enrolled all the subjects in the same season. Another limit of our study was the lack of assessment of sun exposure. However, all the subjects live in the same metropolitan area and, thus, have similar lifestyle habits regarding sun exposure. Although dual X-ray absorptiometry is considered the gold standard to measure body composition, we used BIA to assess body composition given the good relative agreement of BIA with dual X-ray absorptiometry results [41,42]. The latter is a cross-sectional study which is inherently limited in that it cannot establish cause and effect relationships, and our results may not necessarily be valid in non-white populations. 

In conclusion, females had significantly lower 25OHD concentrations than males among the different class of BMI. This sex difference was mainly explained by the higher FM % in women than men. If these findings are also confirmed in other seasons, they may create a background for the revision of vitamin D supplementation dosages. Indeed, future guidelines for monitoring 25OHD concentrations should take into account not only life stages but also sex. Further, a careful assessment of body composition in order to quantify FM % may be warranted in order to adjust vitamin D supplementation dosages. Randomized controlled trials exploring the dose–response effect of vitamin D supplementation according to gender are highly warranted.

## Figures and Tables

**Table 1:** Age, anthropometric characteristics, and 25OHD concentration of the study population according to sex. Vitamin D deficiency was defined as a serum concentration of 25OHD concentration < 20 ng/mL (50 nmol/L), insufficiency between 21 and 29 ng/mL (from 52.5 to 72.5 nmol/L), and normal concentration ≥ 30 ng/mL (75 nmol/L).

Parameters	Males Mean ± SD or Number (%) *n* = 250	Females Mean ± SD or Number (%) *n* = 250	*p*-Value
Age (years)	37.42 ± 11.84	36.58 ± 11.77	0.429
Weight (kg)	103.43 ± 25.00	89.05 ± 23.06	**<0.001**
Height (m)	1.76 ± 0.05	1.64 ± 0.07	**<0.001**
BMI (kg/m^2^)	33.19 ± 7.87	33.01 ± 7.93	0.804
Normal weight	50, 20%	50, 20%	χ^2^ = 0.01, 0.908
Overweight	50, 20%	50, 20%	
Grade I obesity	50, 20%	50, 20%	
Grade II obesity	50, 20%	50, 20%	
Grade III obesity	50, 20%	50, 20%	
25OHD concentration (ng/mL)	19.70 ± 8.31	16.36 ± 7.49	**<0.001**
Deficiency	156, 62.4%	170, 68.0%	χ^2^ = 2.80, ***p* =** 0.094
Insufficiency	50, 20.0%	70, 28.0%	χ^2^ = 4.29, ***p* = 0.038**
Sufficiency	44, 17.6%	10, 4.0%	χ^2^ = 23.31, ***p* = 0.001**

A *p*-value in bold type denotes a significant difference (*p* < 0.05).

**Table 2:** Body composition parameters of the study population assessed by bioelectrical impedance analysis (BIA) reported according to sex.

Parameters	Males *n* = 250	Females *n* = 250	*p*-Value
R (Ω)	467.9 ± 88.9	486.6 ± 84.5	**0.02**
Xc (Ω)	49.2 ± 10.2	46.8 ± 8.9	**0.005**
FM (%)	30.6 ± 14.0	37.5 ± 13.6	**<0.001**
FFM (%)	69.4 ± 14.0	62.5 ± 13.6	**<0.001**
TBW (%)	54.4 ± 10.8	46.7 ± 10.1	**<0.001**
ECW (%)	45.8 ± 3.9	48.2 ± 3.5	**<0.001**
ICW (%)	54.2 ± 3.9	51.8 ± 3.5	**<0.001**

A *p*-value in bold type denotes a significant difference (*p* < 0.05). Fat mass, FM; free fat mass, FFM; total body water, TBW; extra-cellular water, ECW; and intra-cellular water, ICW.

**Table 3:** Correlations of vitamin D with age, BMI, and body composition parameters assessed by BIA according to sex.

Parameters	25OHD Concentration (ng/mL)			
	Males (*n* = 250)		Females (*n* = 250)	
	*r*	*p*-Value	*r*	*p*-Value
Age (years)	−0.002	0.98	−0.08	0.22
BMI (kg/m^2^)	**−0.47**	**<0.001**	**−0.69**	**<0.001**
R (Ω)	**−0.16**	**0.01**	−0.11	0.08
Xc (Ω)	**0.22**	**0.001**	**0.37**	**<0.001**
FM (%)	**−0.45**	**<0.001**	**−0.72**	**<0.001**
FFM (%)	**0.45**	**<0.001**	**0.72**	**<0.001**
TBW (Lt)	**0.41**	**<0.001**	**0.68**	**<0.001**
ECW (Lt)	**−0.52**	**<0.001**	**−0.74**	**<0.001**
ICW (Lt)	**0.52**	**<0.001**	**0.74**	**<0.001**

A *p*-value in bold type denotes a significant difference (*p* < 0.05). R (Ω): resistance; Xc (Ω): reactance; TBW: total body water; ICW: intracellular body water; ECW: extracellular body water; ICW: intracellular body water.

**Table 4:** Multiple regression analysis model (stepwise method) with 25OHD concentration as a dependent variable and sex, BMI, and FM % as independent variables.

Parameters	Multiple Regression Analysis			
	*R* ^2^	β	t	*p*-Value
FM (%)	0.35	0.59	16.5	**<0.001**
BMI (kg/m^2^)	0.36	0.17	−2.4	**0.01**
Sex	0.38	0.13	−3.3	**0.01**

A *p*-value in bold type denotes a significant difference (*p* < 0.05).

## Abbreviations

25OHD	serum concentration serum concentration of vitamin D
BMI	body mass index; BIA, bioelectrical impedance analysis
FM	fat mass
T1DM	type 1 diabetes
T2DM	type 2 diabetes
NHANES	National Health and Nutrition Examination Survey
PTH	parathyroid hormone
HbA1c	glycated hemoglobin
DSM	Diagnostic and Statistical Manual of Mental Disorders
WHO	World Health Organization
kHz	kilohertz
ESPEN	European Society of Parental and Enteral Nutrition
PhA	phase angle
Xc	reactance
R	resistance
CVs	coefficients of variation
FFM	free fat mass
TBW	total body water
ECW	extra-cellular water
ICW	intra-cellular water
